# *Papaver somniferum* and *Papaver rhoeas* Classification Based on Visible Capsule Images Using a Modified MobileNetV3-Small Network with Transfer Learning

**DOI:** 10.3390/e25030447

**Published:** 2023-03-03

**Authors:** Jin Zhu, Chuanhui Zhang, Changjiang Zhang

**Affiliations:** 1 College of Physics and Electronic Information Engineering, Zhejiang Normal University, Jinhua 321000, China; 2 School of Electronics and Information Engineering (School of Big Data Science), Taizhou University, Taizhou 318000, China

**Keywords:** identification, poppy capsule image dataset, MobileNetV3-Small, transfer learning, *Papaver somniferum*, *Papaver rhoeas*

## Abstract

Traditional identification methods for *Papaver somniferum* and *Papaver rhoeas* (PSPR) consume much time and labor, require strict experimental conditions, and usually cause damage to the plant. This work presents a novel method for fast, accurate, and nondestructive identification of PSPR. First, to fill the gap in the PSPR dataset, we construct a PSPR visible capsule image dataset. Second, we propose a modified MobileNetV3-Small network with transfer learning, and we solve the problem of low classification accuracy and slow model convergence due to the small number of PSPR capsule image samples. Experimental results demonstrate that the modified MobileNetV3-Small is effective for fast, accurate, and nondestructive PSPR classification.

## 1. Introduction

The private cultivation of *Papaver somniferum* is illegal in many countries because its extracts can be turned into addictive and poisonous opioids. However, because of the huge profits, the illegal cultivation of *Papaver somniferum* occurs all over the world. The appearance of *Papaver somniferum* is similar to that of its relatives, such as the ornamental plant *Papaver rhoeas*, frequently leading to mistaken identification reports from civilians engaged in anti-drug work. This paper seeks to develop a fast, accurate, and non-destructive identification method for *Papaver somniferum* and its close relatives (represented by *Papaver rhoeas*) to improve civilians’ ability to distinguish between them, thereby effectively assisting the police in drug control work. It also provides model support for the development of *Papaver somniferum* identification systems on mobile terminals.

*Papaver somniferum* is traditionally identified by methods including direct observation, physical and chemical property identification, and spectral analysis. Zhang et al. [1] employed a discrete stationary wavelet transform to extract characteristics from Fourier transform infrared spectroscopy data to identify *Papaver somniferum* and *Papaver rhoeas* (PSPR). Choe et al. [2] used metabolite spectral analysis to identify *Papaver somniferum*, *Papaver rhoeas*, and *Papaver setigerum*. Wang et al. [3] used specific combinations of characteristic wavelength points to distinguish between *Papaver somniferum* and non-poppy plants, proving that spectral properties can be used to identify *Papaver somniferum*. Li [4] used a fluorescent complex amplification test that contained three simple sequence repeats to achieve the precise detection of *Papaver somniferum* and its relatives.

The above methods have limitations that render them unsuitable for the identification of PSPR for ordinary people in daily life. Direct observation, for example, is time-consuming and labor-intensive, and observers must be familiar with the characteristics of these plants. Other approaches require stringent experimental conditions and tedious operations with the potential to harm the plant. In recent years, many scholars have attempted to use aerial images of *Papaver somniferum* to identify *Papaver somniferum* fields, and they have achieved good results. For example, Liu et al. [5] proposed a method based on a single-shot multi-box detector to detect *Papaver somniferum* fields using remote sensing images from the Chinese ZiYuan3 satellite. Wang et al. [6] proposed global multiscale-YOLOv3, based on the YOLOv3 convolutional neural network (CNN), using low-altitude remote sensing images captured by unmanned aerial vehicles (UAVs) to achieve fast and accurate *Papaver somniferum* field detection. However, we found that few scholars have paid attention to the application scenarios for ordinary people identifying *Papaver somniferum* in daily life, though reports from ordinary people are frequent and important in anti-drug work. Because our model is aimed at the general public, models trained from aerial photos of *Papaver somniferum* fields do not meet our requirements. We intend to build a PSPR dataset by collecting PSPR visible capsule images and use image classification technology based on deep learning to achieve direct and effective identification. We chose the capsule images of PSPR for identification because the capsule stage of *Papaver somniferum* is more harmful. The extracts used to make addictive and poisonous opioids are mainly obtained from the capsules.

Hinton et al. [7] defined deep learning as a network structure with several hidden layers and numerous perceptrons that automatically extracts and combines low-level characteristics to construct abstract high-level features or attribute classes to discover distributed feature representations of data [8]. Since the breakthrough of AlexNet [9] in the field of massive image classification, the use of CNNs to classify images has become a popular research topic in the field of computer vision. Compared with traditional image classification algorithms, the CNN avoids the manual extraction of target image features, and instead autonomously learns more abstract levels of image features through deep architecture, and the extracted features are closely related to the classifier [10], resulting in better generalization.

To better extract image features and improve classification accuracy, scholars have explored deeper and wider networks to improve the learning ability of models and proposed deep convolutional neural network (DCNN) models such as GoogleNet [11], VGGNet [12], and ResNet [13]. The DCNNs above have achieved better accuracy and robustness on the ImageNet dataset, and the performance of the network models has been continuously improved. However, their complexity and number of parameters have increased tremendously. VGGNet-16, for example, uses almost 138 million parameters and 15 giga floating point operations (FLOPs).

The characteristics of DCNNs impose great constraints on the computation, memory space, portability, and energy consumption of a device. There is increasing demand for mobile terminals, and combining them with deep learning technology has become an important trend [14]. The idea of lightweight neural networks has been proposed in this context, and two research ideas have been proposed to solve the obstacles of neural networks from laboratory to application [15]. One is to accelerate and compress high-performance models using compression algorithms such as parameter pruning and knowledge distillation [16], and the other is to design and build efficient lightweight network models. The goal of lightweight networks is to achieve the best balance between the accuracy, size, and speed of the model.

The lightweight CNN model, such as SqueezeNet [17], the ShuffleNet series [18,19], the MobileNet series [20,21,22], and GhostNet [23], are widely used in the field of image classification. Cui et al. [24] used a compact and efficient spatial attention module based on MobileNetV3 to considerably minimize computation and model parameters while maintaining good hyperspectral image classification accuracy. Chen et al. [25] proposed a lightweight garbage classification network based on ShuffleNetV2, with a parallel mixed attention mechanism and FReLU activation function. Liu et al. [26] improved the SqueezeNet network using training optimization strategies such as MSRA initialization, RMSprop, and the momentum method for the classification of food crops. Wei et al. [27] updated GhostNet to increase classification accuracy and reduce intermediate parameters, according to the features of remote sensing image datasets, and they achieved higher classification accuracy on the AID, UC Merced, and NWPU-RESISC45 datasets.

DCNNs can show superior performance only when there are enough training samples. They are prone to phenomena such as overfitting and slipping into local optima when training samples are insufficient [9]. Because *Papaver somniferum* cultivation is strictly controlled by the government, it is difficult to obtain training data with numerous samples of *Papaver somniferum* capsule images, and because there is no publicly available PSPR capsule dataset, we can only rely on an Internet image search to build our experimental dataset, which results in a small sample. Transfer learning is a useful machine learning method that applies the knowledge or patterns learned in a certain domain or task to a different but related domain or problem. Existing feature extraction capabilities can be leveraged to accelerate and optimize model learning efficiency with the parameters of a neural network model trained on a large image dataset transferred to a target model to aid in the training of a new model, enabling the training of models with higher recognition accuracy using smaller training samples [28]. Transfer learning can effectively improve the accuracy and robustness of the model, and has been widely used in text processing, [29,30,31] image classification [32,33,34], collaborative filtering [35,36,37], and artificial intelligence planning [38,39].

MobileNetV3 has the advantages of high classification accuracy and a fast classification speed, and it can better balance efficiency and accuracy for image classification on mobile devices. We propose a new classification model, P-MobileNet, based on an improved MobileNetV3 network with transfer learning from ImageNet. This study provides a new solution for fast, accurate, and nondestructive identification of PSPR for ordinary people, and it can be extended to identify any relatives of *Papaver somniferum*.

The main contributions of this paper are as follows:

- A database of 1496 *Papaver somniferum* capsule images and 1325 *Papaver rhoeas* capsule images is established;
- The structure of the MobileNetV3 network is improved to reduce the number of parameters and amount of computation, achieving fast, convenient, accurate, and non-destructive identification of PSPR;
- The effectiveness of data expansion and transfer learning for model training is experimentally verified, and the influence of different transfer learning methods on the model is compared;
- The improved MobileNetV3 model combined with transfer learning solves the problem of low classification accuracy and slow model convergence due to the small number of PSPR capsule image samples, and it improves the robustness and classification accuracy of the proposed classification model.

## 2. Data

**Figure 1:**
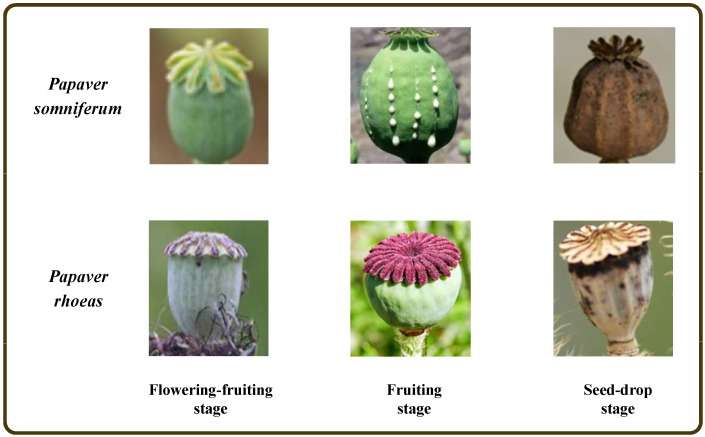
Capsule images of PSPR at different capsule developmental stages.

It is difficult to take images of *Papaver somniferum* capsules in the field because its cultivation is strictly controlled by the government. Therefore, all datasets for this experiment were collected from an Internet search, with a total of 2821 images, comprising 1496 images of *Papaver somniferum* capsules and 1325 images of *Papaver rhoeas* capsules. The intercepted images were taken under different angles and light, and covered all growth and development stages of the capsule stage (flowering-fruiting, fruiting, and seed-drop), as shown in Figure 1. Note that the capsule images in the dataset are not of the same size and are resized consistently in Figure 1 for aesthetics. The maximum and minimum sizes of images in the capsule dataset are 624×677 pixels and 27×35 pixels, respectively.

**Figure 2:**
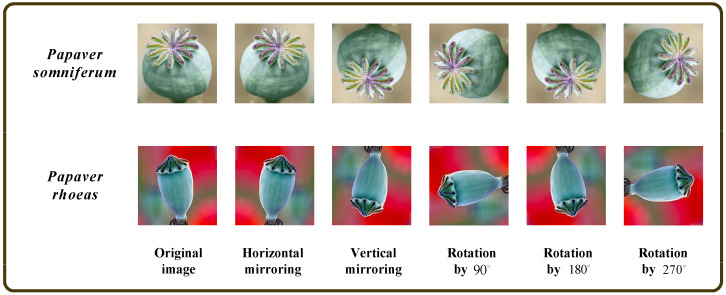
Capsule image training set of PSPR using data expansion.

The establishment of the PSPR capsule image dataset can be divided into the following steps:

1. First, the dataset was mixed and scrambled and separated into training, validation, and testing data at a ratio of 8:1:1;
2. To improve the model’s feature-extraction and generalization ability and avoid the problems of overfitting and low classification accuracy caused by a small sample dataset, the capsule image training set was expanded using common data expansion methods in deep learning [28], that is, horizontal mirroring, vertical mirroring, and rotation by 90, 180, and 270 degrees, respectively, as shown in Figure 2. As in Figure 1, the capsule images in Figure 2 are resized to a consistent size. The expanded training set includes 7170 *Papaver somniferum* capsule images and 6366 *Papaver rhoeas* capsule images;
3. Finally, all image sizes were resized to 224×224 pixels to ensure that the data suited the model’s input size.

**Figure 3:**
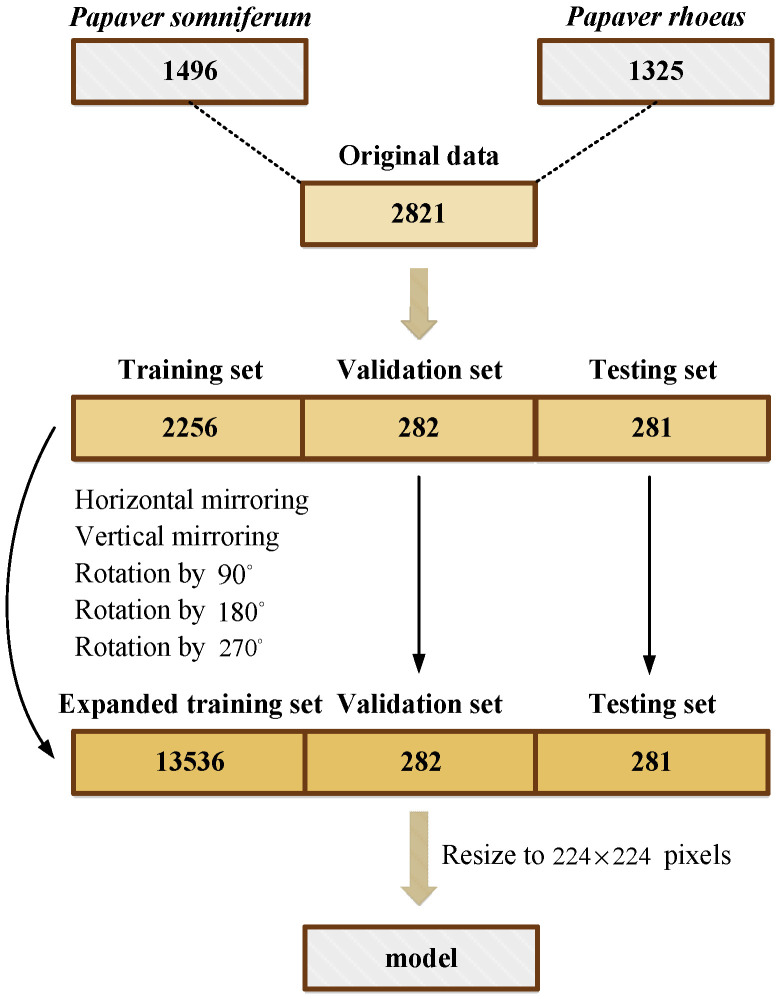
Establishment of the capsule image dataset of PSPR.

The process flow of the establishment of the capsule image dataset is shown in Figure 3.

## 3. Methods

### 3.1. Basic MobileNetV3-Small

**Figure 4:**
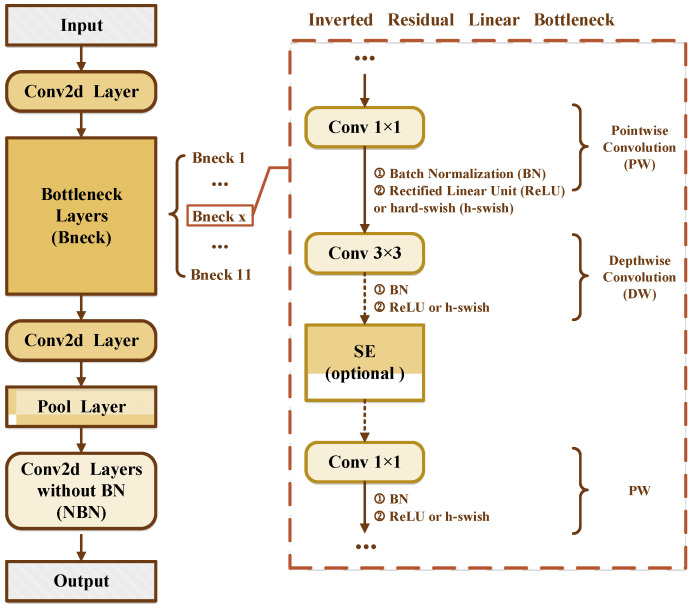
MobileNetV3 structure.

MobileNetV3, as part of a new generation of lightweight networks, builds on MobileNetV1 and MobileNetV2 by combining deep separable convolution and an inverse residual structure with a linear bottleneck to improve computational efficiency and effectively extract feature information. It uses platform-aware Neural Architecture Search [40] and Neural Network Adaptation [41] to optimize the network structure and parameters. A Squeeze-and-Excite (SE) [24] channel attention module further improves network performance and operational efficiency. Figure 4 shows the MobileNetV3 structure.

MobileNetV3 includes two versions: MobileNetV3-Small and MobileNetV3-Large, with similar architecture but different complexity to suit different scenarios. MobileNetV3-Small is suitable for low-performance mobile devices and embedded devices. Considering the issues of computational cost and model efficiency, we use MobileNetV3-Small as the basic framework of the PSPR classifier and improve its network structure.

### 3.2. Construction of Network for Papaver Somniferum Identification

**Figure 5:**
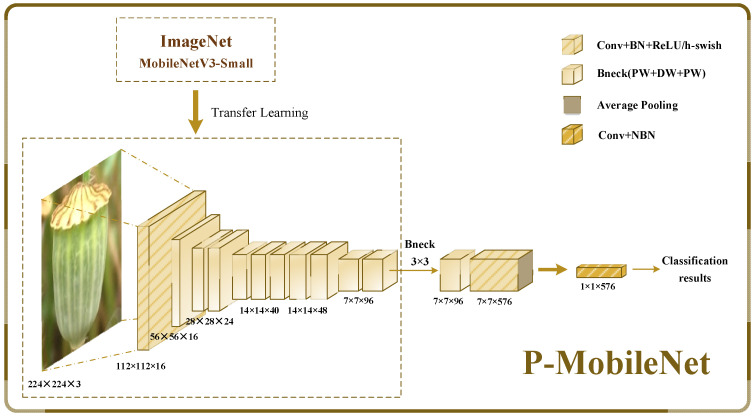
P-MobileNet model structure.

We propose a P-MobileNet model based on transfer learning and a modified MobileNetV3-Small model to lower the model’s data requirements while improving operational efficiency. Figure 5 shows the P-MobileNet model structure, which consists of a pre-trained MobileNetV3-Small model on the ImageNet dataset and a modified MobileNetV3-Small model.

#### 3.2.1. Transfer Learning

DCNNs often fail to achieve higher prediction performance with small sample datasets, they are prone to problems such as training difficulty and overfitting [9], and it is sometimes difficult to obtain a large amount of data with labels. Transfer learning is an efficient strategy to solve image classification problems with small samples [32,33,34,42,43].

There are two main approaches for applying a pre-trained DCNN to a new image classification task [9,44]. One approach, called transfer learning method 1 (TL_M1), is to freeze all the weights of the convolutional layers from the pre-trained model and use them as fixed-feature extractors [9,45,46], and fully connected layers are added and trained using the new sample dataset. The other, called transfer learning method 2 (TL_M2), is to initialize the target model using the weights of the pre-trained model and then fine-tune the network weights training on the new sample dataset [9,47,48]. The impact of transfer learning on the model will be described in detail in Section 4.3 through experiments.

#### 3.2.2. Modified MobileNetV3-Small Model

MobileNetV3-Small performed well on the challenging thousand-classification task on ImageNet. As for our binary identification task, deep networks impose excessive calculation costs and affect the classification speed. Consequently, after the analysis of the network configuration, we modified the architecture of the MobileNetV3-Small network to improve efficiency without degrading performance. The kernel size of the depthwise convolution of the last bottleneck layer of the original MobileNetV3-Small model is modified from 5×5 to 3×3 to reduce the calculation and latency of feature extraction. The last two 1×1 convolution layers, responsible for extrapolation and classification, are reduced to one layer to reduce the number of parameters. These changes significantly reduce the number of model parameters, along with the computational burden, while maintaining accuracy. Table 1 shows the network structure of the proposed P-MobileNet model.

The columns in Table 1 are as follows: (1) Input represents the feature map size input to each feature layer of MobileNetV3; (2) Operator represents the layer structure which each feature map will cross; (3) Exp size represents the number of channels after the inverse residual structure in the bottleneck rises; (4) Out represents the number of channels in the feature map after passing the bottleneck; (5) SE represents whether the SE attention mechanism is introduced at this layer; (6) NL represents the type of activation function used, HS (h-swish) or RE (ReLU); and (7) S represents the step size used for each layer structure.

## 4. Experimental Results and Discussion

### 4.1. Experimental Environment

The configuration used for model training and testing in this paper is as follows: Intel Core i5-10210U CPU @ 1.60 GHz/2.11 GHz; 16 GB RAM; Nvidia GeForce MX250 graphics card; Windows 10 Home Chinese version; CUDA version 10.1; and PyTorch 3.8.

### 4.2. Evaluation Indicators

The model was evaluated based on accuracy, *precision* (*P*), *recall* (*R*), *F*1, number of parameters, computation (measured using FLOPs), weight file size, and average prediction time for a single image. The task of PSPR is a binary classification problem, and we define *Papaver somniferum* as the positive class and *Papaver rhoeas* as the negative class.

*Accuracy*, *precision*, *recall*, and *F*1 are defined as follows [6,49]:

(1) $$\;accuracy=\frac{TP+TN}{TP+FP+TN+FN}$$

(2) $$precision=\frac{TP}{TP+FP}$$

(3) $$recall=\frac{TP}{TP+FN}$$

(4) $$F1=\frac{2PR}{P+R}$$

where *TP* (true positive) is the real examples, *FN* (false negative) is the false negative examples, *FP* (false positive) is the false positive examples, and *TN* (true negative) is the true negative examples.

*Accuracy* reflects the proportion of correct predictions in the entire sample; *Precision* reflects the proportion of samples with positive predictions that are positive; *Recall* indicates the proportion of all positive samples that are correctly predicted; *F*1 is the summed average of *precision* and *recall* [25].

### 4.3. Experiments on Influencing Factors of Model Performance

#### 4.3.1. Experimental Design

The MobileNetV3-Small model trained on the ImageNet dataset was selected as the basic model and P-MobileNet was the target model. Six sets of experiments were conducted, combined with three learning methods (training from scratch, TL_M1, and TL_M2) and two data expansion methods (unexpanded data and expanded data).

Specifically, training from scratch means randomly initializing the weight parameters of all layers of the model, and the capsule image dataset is used to train the model, following which the back-propagation algorithm is used to tune its weights. In TL_M1, the pre-trained model’s weights are used as fixed feature extractors and the linear classifiers are trained on the new sample dataset. To clarify, since the feature extraction layer structure of P-MobileNet is not identical to that of MobileNetV3-Small (the kernel size of the depthwise convolution of the last bottleneck layer of MobileNetV3-Small is modified from 5×5 to 3×3), the weight information of this layer is not passed from the pre-trained model but is trained from scratch together with the classification layer (which is a 1×1 convolutional layer without batch normalization in P-MobileNet). In TL_M2, the new sample dataset is used to fine-tune all layers of the model initialized by the weights of the pre-trained model (the weight information of the last bottleneck layer from the pre-trained model is ignored, as in TL_M1). This enables the model to learn highly generalizable features from a larger sample dataset, while the features are more relevant to the new classification task.

Regarding the data expansion methods, training under unexpanded data means the model is trained using the original capsule image dataset with 2821 images, while the other is training under the expanded capsule image dataset with 14,099 images, using the data expansion method described in Section 2.

Considering the computation and training time, the batch size for both testing and training was set to eight. The Adam optimizer was used with a learning rate of 0.0001, and the maximum number of training rounds was set to 120 epochs.

#### 4.3.2. Experimental Results and Analysis

After 120 training epochs, a comparison of the performance of P-MobileNet under different learning methods and data expansion methods is shown in Table 2. In addition to the accuracy, precision, recall, and F1 values of the testing set, we also calculated the standard deviation (SD) of the training loss (train_loss) and the accuracy of the validation set (val_acc) to measure the volatility of the data.

1. Influence of different learning methods on model performance.

**Figure 6:**
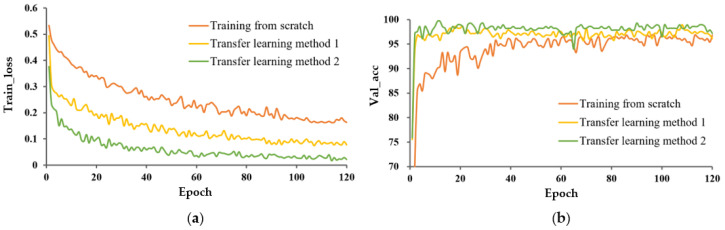
Performance evaluation of P-MobileNet trained on original capsule image dataset. (**a**) Loss function curve on the training set and (**b**) Accuracy of the validation set.

**Figure 7:**
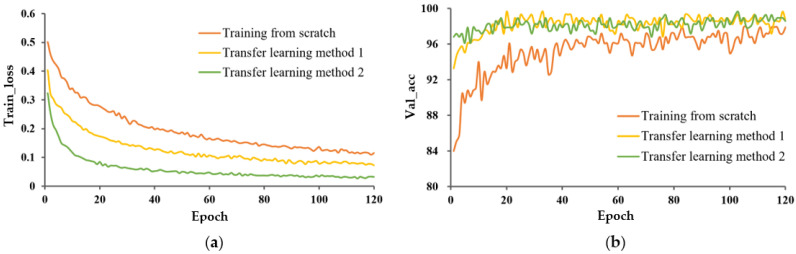
Performance evaluation of P-MobileNet trained on expanded capsule image dataset. (**a**) Loss function curve on the training set and (**b**) Accuracy curve on the validation set.

The train_loss curve and val_acc for the three learning methods are shown in Figure 6 and Figure 7, respectively. In both cases, P-MobileNet trained from scratch had the slowest convergence rate with large fluctuations, and the loss function presented a high loss value after stabilization. The model with TL_M2 had the fastest convergence speed and lowest loss value. The accuracy of P-MobileNet trained from scratch was the lowest and fluctuated greatly. The accuracy of the model with transfer learning fluctuated less, among which the accuracy of TL_M2 was the highest. The SD of val_acc for TL_M2 under unexpanded data was decreased by 3.354 percentage points compared to that for training from scratch.

The differences in the model performance between TL_M1 and TL_M2 were relatively small, but it can still be observed that P-MobileNet with TL_M2 was more advantageous than training with TL_M1. From Table 2, the F1 value of TL_M2 was more than 1 percentage point higher than that of TL_M1, which shows that P-MobileNet with TL_M2 has higher recognition accuracy and robustness.

These results indicate that transfer learning effectively solved the problems of low classification accuracy and slow model convergence due to a small-sample dataset.

2. Effect of data expansion on model performance.

**Figure 8:**
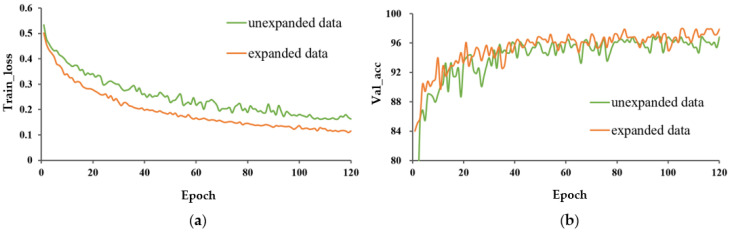
Performance evaluation of P-MobileNet trained from scratch. (**a**) Loss function curve on the training set and (**b**) Accuracy curve on the validation set.

**Figure 9:**
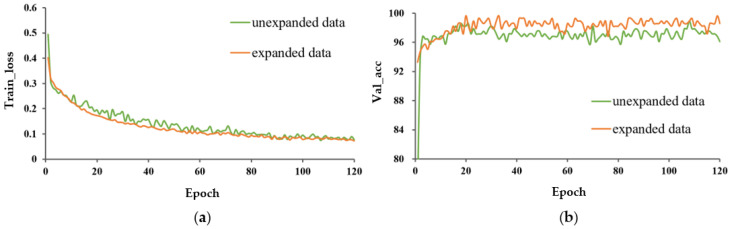
Performance evaluation of P-MobileNet with TL_M1. (**a**) Loss function curve on the training set and (**b**) Accuracy curve on the validation set.

**Figure 10:**
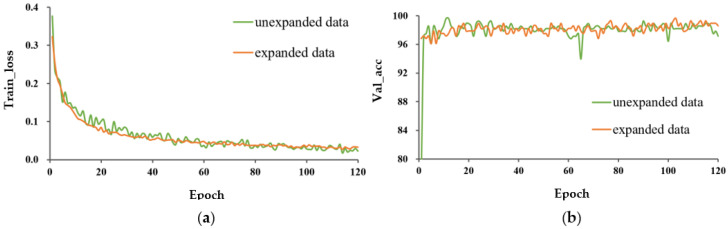
Performance evaluation of P-MobileNet with TL_M2. (**a**) Loss function curve on the training set and (**b**) Accuracy curve on the validation set.

The train_loss and val_acc for the expanded and unexpanded datasets under three different training methods are shown in Figure 8, Figure 9 and Figure 10. For these three different learning methods, a general phenomenon was observed, namely that the loss function of the model trained on the expanded capsule image dataset was lower and less volatile than that on the original dataset. From Table 2, for training from scratch, TL_M1, and TL_M2, the test accuracy under expanded data was 1.4, 0.7, and 0.3 percentage points higher, respectively, than that for training on the original data; the SD of val_acc under expanded data was decreased by 2.924, 1.115, and 1.429 percentage points compared to that trained on the original data, respectively, which indicated that data expansion could improve the classification accuracy and robustness of the model.

It could also be found that, under the model trained from scratch, data expansion had a greater promotion effect on improving the accuracy of the model and avoiding the phenomenon of overfitting than under the model with transfer learning. This was mainly due to the fact that the pre-trained model learned a large amount of knowledge on the large image dataset, weakening the role of data expansion.

In any case, the accuracy and robustness of the model were improved by different magnitudes on the expanded capsule image dataset, regardless of the learning strategy, indicating that the data expansion provided the necessary amount of data for model training and that a certain size of dataset is still necessary.

To summarize, the expanded capsule image dataset was used to train P-MobileNet with TL_M2.

### 4.4. Comparison of Classification Networks

To verify the effectiveness of P-MobileNet for PSPR identification, we compared various DCNNs on the self-constructed PSPR capsule image dataset (including the expanded training data, unexpanded validation data, and test data), with a total of 14099 images. Models included some representative traditional CNNs (AlexNet, GoogLeNet, ResNet-34) and popular lightweight networks. All models were trained under transfer learning. Classification results were compared in terms of accuracy, precision, recall, F1, number of parameters, FLOPs, weight file size, and average prediction time for a single image on the testing set, as shown in Table 3.

Table 3 further illustrates that the traditional network models could not meet the requirements for mobile deployment because of their enormous calculations. Lightweight networks tend to have much fewer parameters and FLOPs than traditional networks, but they have comparable or even better model performance. Among the lightweight network models, SqueezeNet had the fewest parameters and smallest model size but the lowest accuracy and recall rates, 96.2% and 94.7%, respectively. ShuffleNetV2 outperformed SqueezeNet, with the smallest FLOPs of 2.28 M, but the largest number of parameters, 148.8 M. The performance of GhostNet and MobileNetV3 exceeded that of ResNet-34.

MobileNetV3 performed best. The number of parameters, amount of computation, and model size of MobileNetV3-Small were much smaller than those of MobileNetV3-Large, while they showed similar performance at PSPR classification, which further indicates the redundancy of the MobileNetV3 model for this task. Compared with MobileNetV3-Small, the recall of P-MobileNet increased by 0.8 percentage points, and the F1 value was the same, at 98.9%. However, P-MobileNet had only 36% of the parameters of MobileNetV3-Small, and it used less calculation. The model was only slightly larger than SqueezeNet, and the prediction speed was the fastest.

**Figure 11:**
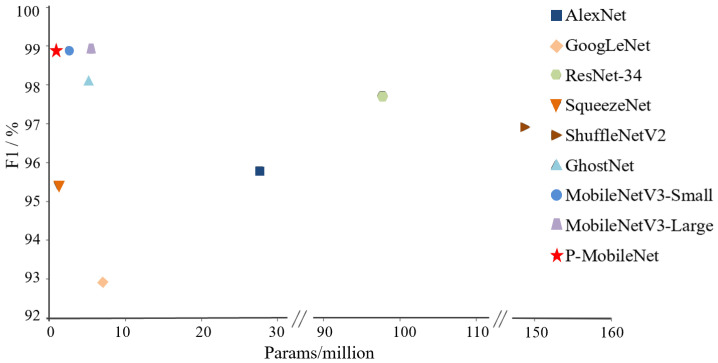
Relationship between F1 and total model parameters.

We compared the performance of the models based on F1 and the number of parameters, as shown in Figure 11, where the horizontal scale is the number of parameters and the vertical scale is F1. P-MobileNet had the highest F1 with the fewest parameters.

Based on these results, P-MobileNet best balanced accuracy and efficiency for the PSPR classification task, with a classification accuracy of 98.9% and an average prediction time of 45.7 ms for a single image, which is better than other tested models.

## 5. Conclusions

The appearance of *Papaver somniferum* is similar to that of *Papaver rhoeas*, increasing the difficulty of its identification. Traditional methods of *Papaver somniferum* identification, including direct observation, physical and chemical property identification, and spectral analysis, cannot be applied to drug-related cases and *Papaver somniferum* identification in daily life. To solve these problems, we proposed the P-MobileNet model for PSPR classification, based on the improved MobileNetV3-Small with transfer learning.

- Compared with training from scratch, transfer learning could fully utilize the knowledge learned on large datasets, significantly accelerated the convergence speed of the model, and improved the classification performance. Regardless of the type of transfer learning method adopted, pre-training and fine-tuning P-MobileNet had a superior impact than that obtained by training P-MobileNet from scratch. The feature extraction ability of the random initialization model was not good enough under a small sample dataset;
- The impact of data expansion on the model trained from scratch was greater than that of the model with transfer learning. Data expansion enriched the diversity of data, which was helpful to mitigate overfitting and improved the classification performance of the model. Although transfer learning weakened the effect of data expansion, a certain amount of training set expansion was necessary to improve the robustness of the model;
- Analysis of the classification performance of different models showed that the proposed P-MobileNet model has the advantages of high classification accuracy, a few parameters, and a fast detection speed. Compared with MobileNetV3-Small, P-MobileNet maintains a high classification accuracy of 98.9%, with only 36% of the parameters of the MobileNetV3-Small model; the FLOPs are reduced by 2 M; and the detection speed is improved to 45.7 ms/image. This study provides a means to achieve the rapid, accurate, and non-destructive identification of PSPR on mobile terminals.

## Figures and Tables

**Table 1:** The network structure of the P-MobileNet model.

Input	Operator	Exp Size	Out	SE	NL	S
$224^{2}\times 3$	Conv2d, 3×3	-	16	-	HS	2
$112^{2}\times 16$	Bneck, 3×3	16	16	√	RE	2
$56^{2}\times 16$	Bneck, 3×3	72	24	-	RE	2
$28^{2}\times 24$	Bneck, 3×3	88	24	-	RE	1
$28^{2}\times 24$	Bneck, 5×5	96	40	√	HS	2
$14^{2}\times 40$	Bneck, 5×5	240	40	√	HS	1
$14^{2}\times 40$	Bneck, 5×5	240	40	√	HS	1
$14^{2}\times 40$	Bneck, 5×5	120	48	√	HS	1
$14^{2}\times 48$	Bneck, 5×5	144	48	√	HS	1
$14^{2}\times 48$	Bneck, 5×5	288	96	√	HS	2
$7^{2}\times 96$	Bneck, 5×5	576	96	√	HS	1
$7^{2}\times 96$	Bneck, 3×3	576	96	√	HS	1
$7^{2}\times 96$	Conv2d, 1×1	-	576	√	HS	1
$7^{2}\times 576$	Pool, 7×7	-	-	-	-	1
$1^{2}\times 576$	Conv2d 1×1, NBN	-	2	-	-	1

**Table 2:** Performance comparison of P-MobileNet under different learning methods and data expansion.

Learning Method	Data Expansion	Accuracy/%	Precision/%	Recall/%	F1/%	SD of Train_Loss	SD of Val_Acc
Training from scratch	×	95.0	94.7	94.7	94.7	0.083	5.430
	√	96.4	97.7	94.7	96.2	0.081	2.506
TL_M1	×	97.2	97.7	96.2	96.9	0.063	2.150
	√	97.9	98.5	97.0	97.7	0.059	1.035
TL_M2	×	98.6	99.2	97.7	98.5	0.050	2.076
	√	98.9	99.2	98.5	98.9	0.045	0.647

**Table 3:** Performance comparison of classification networks on the testing set.

Model	Accuracy/%	Precision/%	Recall/%	F1/%	Params/Million (M)	FLOPs/Million (M)	Model Size /MB	Test Time /ms
AlexNet	96.1	95.5	96.2	95.8	27.6	681.2	97.4	38.6
GoogLeNet	93.2	91.9	93.9	92.9	7.0	1624.1	39.3	54.6
ResNet-34	97.9	100	95.5	97.7	97.7	3759.1	81.3	88.6
SqueezeNet	95.7	96.2	94.7	95.4	1.2	351.9	2.8	48.6
ShuffleNetV2	97.2	97.7	96.2	96.9	148.8	2.3	5.0	50.3
GhostNet	98.2	99.2	97.0	98.1	5.2	148.8	15.1	48.6
MobileNetV3-Small	98.9	100	97.7	98.9	2.5	59.4	5.9	47.5
MobileNetV3-Large	98.9	100	97.7	98.9	5.5	225.4	16.2	48.2
**P-MobileNet**	**98.9**	**99.2**	**98.5**	**98.9**	**0.9**	**57.3**	**3.6**	**45.7**

## Data Availability

The datasets used and/or analyzed during the current study are available from the corresponding author upon reasonable request.

## References

[1] Zhang C.-J., Cheng C.-G. (2009). Identification of *Papaver Somniferum* L. and *Papaver Rhoeas* Using DSWT-FTIR-RBFNN. Spectrosc. Spect. Anal..

[2] Choe S., Kim S., Lee C., Yang W., Park Y., Choi H., Chung H., Lee D., Hwang B.Y. (2011). Species identification of Papaver by metabolite profiling. Forensic Sci. Int..

[3] Wang H., Qin L.-A., Jing X., He F., Tan F.-F., Hou Z.-H. (2019). Research of identification of papaver based on spectral analysis. Chin. J. Quantum Electron.

[4] Li Y.-Y. (2016). Construction and Application of a Fluorescent Complex Amplification System for Three Poppy SSR Motifs. Master’s Thesis.

[5] Liu X., Tian Y., Yuan C., Zhang F., Yang G. (2018). Opium poppy detection using deep learning. Remote Sens..

[6] Wang C., Wang Q., Wu H., Zhao C., Teng G., Li J. (2021). Low-altitude remote sensing opium poppy image detection based on modified yolov3. Remote Sens..

[7] Hinton G.E., Salakhutdinov R.R. (2006). Reducing the dimensionality of data with neural networks. Science.

[8] Wei J., Liu A.-J., Tang J.-W. (2017). Research on alarm model of digital TV monitoring platform based on deep learning neural network technology. Cable Telev. Technol..

[9] Krizhevsky A., Sutskever I., Hinton G.E. (2017). Imagenet classification with deep convolutional neural networks. Commun. ACM..

[10] Yang Z.-Z., Kuang N., Fan L., Kang B. (2018). A Review of Image Classification Algorithms Based on Convolutional Neural Networks. J. Signal Process..

[11] Szegedy C., Liu W., Jia Y., Sermanet P., Reed S., Anguelov D., Erhan D., Vanhoucke V., Rabinovich A. Going deeper with convolutions. Proceedings of the IEEE Conference on Computer Vision and Pattern Recognition.

[12] Simonyan K., Zisserman A. (2014). Very deep convolutional networks for large-scale image recognition. arXiv.

[13] He K., Zhang X., Ren S., Sun J. Deep residual learning for image recognition. Proceedings of the IEEE Conference on Computer Vision and Pattern Recognition.

[14] Hao L., Weigen Q., Lichen Z. (2022). Improved ShuffleNet V2 for Lightweight Crop Disease Identification. Comput. Eng. Appl..

[15] Zhang X., Zou J., He K., Sun J. (2015). Accelerating very deep convolutional networks for classification and detection. ITPAM.

[16] Han S., Mao H., Dally W.J. (2015). Deep compression: Compressing deep neural networks with pruning, trained quantization and huffman coding. arXiv.

[17] Iandola F.N., Han S., Moskewicz M.W., Ashraf K., Dally W.J., Keutzer K. (2016). SqueezeNet: AlexNet-level accuracy with 50x fewer parameters and< 0.5 MB model size. arXiv.

[18] Zhang X., Zhou X., Lin M., Sun J. ShuffleNet: An Extremely Efficient Convolutional Neural Network for Mobile Devices. Proceedings of the 2018 IEEE/CVF Conference on Computer Vision and Pattern Recognition.

[19] Ma N., Zhang X., Zheng H.T., Sun J., Ferrari V., Hebert M., Sminchisescu C., Weiss Y. (2018). ShuffleNet V2: Practical Guidelines for Efficient CNN Architecture Design. Computer Vision—ECCV 2018.

[20] Howard A.G., Zhu M., Chen B., Kalenichenko D., Wang W., Weyand T., Andreetto M., Adam H. (2017). Mobilenets: Efficient convolutional neural networks for mobile vision applications. arXiv.

[21] Sandler M., Howard A., Zhu M., Zhmoginov A., Chen L.-C. (2018). MobileNetV2: Inverted Residuals and Linear Bottlenecks. arXiv.

[22] Howard A., Sandler M., Chu G., Chen L.C., Chen B., Tan M., Wang W., Zhu Y., Pang R., Vasudevan V. (2019). Searching for MobileNetV3. arXiv.

[23] Han K., Wang Y.H., Tian Q., Guo J.Y., Xu C.J., Xu C. GhostNet: More Features from Cheap Operations. Proceedings of the IEEE Conference on Computer Vision and Pattern Recognition (CVPR).

[24] Cui Y., Xia J., Wang Z., Gao S., Wang L. (2022). Lightweight Spectral–Spatial Attention Network for Hyperspectral Image Classification. ITGRS.

[25] Chen Z., Yang J., Chen L., Jiao H. (2022). Garbage classification system based on improved shufflenet v2. Resour. Conserv. Recycl..

[26] Liu Y., Zhao Z., Zhu J., Shen Z., Sun L. A Classification Algorithm of Grain Crop Image Based on Improved SqueezeNet Model. Proceedings of the 2021 IEEE 3rd International Conference on Frontiers Technology of Information and Computer (ICFTIC).

[27] Wei B., Shen X., Yuan Y. (2020). Remote sensing scene classification based on improved Ghostnet. Proceedings of the International Conference on Computer Science and Communication. The Journal of Physics: Conference Series.

[28] Yang H.-Y., Xiao X.-M., Huang Q., Zheng G.-L., Yi W.-L. (2022). Rice Pest Identification Based on Convolutional Neural Network and Transfer Learning. Laser Optoelectron. Prog..

[29] Howard J., Ruder S. (2018). Universal language model fine-tuning for text classification. arXiv.

[30] Peters M., Neumann M., Iyyer M., Gardner M., Clark C., Lee K., Zettlemoyer L. (2018). Deep contextualized word representations. arXiv.

[31] Bataa E., Wu J. (2019). An investigation of transfer learning-based sentiment analysis in Japanese. arXiv.

[32] Kumar S., Janet B. (2022). DTMIC: Deep transfer learning for malware image classification. J. Inf. Secur. Appl..

[33] Lu X., Sun X., Diao W., Feng Y., Wang P., Fu K. (2021). LIL: Lightweight incremental learning approach through feature transfer for remote sensing image scene classification. ITGRS.

[34] Peng L., Liang H., Li T., Sun J. (2021). Rethink Transfer Learning in Medical Image Classification. arXiv.

[35] Pan W., Liu M., Ming Z. (2016). Transfer learning for heterogeneous one-class collaborative filtering. IEEE Intell. Syst..

[36] Cai W., Zheng J., Pan W., Lin J., Li L., Chen L., Peng X., Ming Z. (2019). Neighborhood-enhanced transfer learning for one-class collaborative filtering. Neurocomputing.

[37] Chen X., Pan W., Ming Z. Adaptive Transfer Learning for Heterogeneous One-Class Collaborative Filtering. Proceedings of the 2020 International Joint Conference on Neural Networks (IJCNN).

[38] Zhuo H.H., Yang Q. (2014). Action-model acquisition for planning via transfer learning. Artif. Intell..

[39] Yu L., Shao X., Wei Y., Zhou K. (2018). Intelligent land-vehicle model transfer trajectory planning method based on deep reinforcement learning. Sensors.

[40] Zoph B.A.L., Quoc V. (2016). Neural Architecture Search with Reinforcement Learning. arXiv.

[41] Yang T.J., Howard A., Chen B., Zhang X., Go A., Sandler M., Sze V., Adam H. (2018). NetAdapt: Platform-Aware Neural Network Adaptation for Mobile Applications. arXiv.

[42] Zhuang F., Qi Z., Duan K., Xi D., Zhu Y., Zhu H., Xiong H., He Q. (2020). A comprehensive survey on transfer learning. Proc. IEEE.

[43] Gan J., Qi L., Qin C., He G. (2019). Lightweight fingerprint classification model combined with transfer learning. J. Image Graph..

[44] Wang M., Zhuang Z., Wang K., Zhou S., Liu Z. (2021). Intelligent classification of ground-based visible cloud images using a transfer convolutional neural network and fine-tuning. OExpr.

[45] Razavian A.S., Azizpour H., Sullivan J., Carlsson S. CNN Features Off-the-Shelf: An Astounding Baseline for Recognition. Proceedings of the 2014 IEEE Conference on Computer Vision and Pattern Recognition Workshops.

[46] Penatti O.A.B., Nogueira K., Santos J.A.D. Do deep features generalize from everyday objects to remote sensing and aerial scenes domains?. Proceedings of the 2015 IEEE Conference on Computer Vision and Pattern Recognition Workshops (CVPRW).

[47] Donahue J., Jia Y., Vinyals O., Hoffman J., Zhang N., Tzeng E., Darrell T. DeCAF: A Deep Convolutional Activation Feature for Generic Visual Recognition, Eric, P.X., Tony, J., Eds. Proceedings of the 31st International Conference on Machine Learning.

[48] Azizpour H., Sharif Razavian A., Sullivan J., Maki A., Carlsson S. From generic to specific deep representations for visual recognition. Proceedings of the IEEE Conference on Computer Vision and Pattern Recognition Workshops.

[49] Yu X., Kang C., Guttery D.S., Kadry S., Chen Y., Zhang Y.D. (2021). ResNet-SCDA-50 for Breast Abnormality Classification. IEEE/ACM Trans. Comput. Biol. Bioinform..

